# Customizable, conformal, and stretchable 3D electronics via predistorted pattern generation and thermoforming

**DOI:** 10.1126/sciadv.abj0694

**Published:** 2021-10-13

**Authors:** Jungrak Choi, Chankyu Han, Seokjoo Cho, Kyuyoung Kim, Junseong Ahn, Dionisio Del Orbe, Incheol Cho, Zhi-Jun Zhao, Yong Suk Oh, Hyunsoo Hong, Seong Su Kim, Inkyu Park

**Affiliations:** 1 Department of Mechanical Engineering, Korea Advanced Institute of Science and Technology (KAIST), 291 Daehak-ro, Yuseong-gu, Daejeon 34141, South Korea.; 2 Department of Nano Manufacturing Technology, Korea Institute of Machinery and Materials (KIMM),156 Gajeongbuk-ro, Yuseong-gu, Daejeon 34103, South Korea.; 3 KAIST Institute (KI) for the NanoCentury, Korea Advanced Institute of Science and Technology (KAIST), Daejeon 34141, South Korea.

## Abstract

Recently, three-dimensional electronics (3DE) is attracting huge interest owing to the increasing demands for seamless integration of electronic systems on 3D curvilinear surfaces. However, it is still challenging to fabricate 3DE with high customizability, conformability, and stretchability. Here, we present a fabrication method of 3DE based on predistorted pattern generation and thermoforming. Through this method, custom-designed 3DE is fabricated through the thermoforming process. The fabricated 3DE has high 3D conformability because the thermoforming process enables the complete replication of both the overall shape and the surface texture of the 3D mold. Furthermore, the usage of thermoplastic elastomer and a liquid metal–based conductive electrode allows for high thermoformability during the device fabrication as well as high stretchability during the device operation. We believe that this technology can enable a wide range of new functionalities and multiscale 3D morphologies in wearable electronics.

## Teaser

Thermoforming is a versatile technique to fabricate stretchable and customizable 3DEs for a variety of wearable applications.

## INTRODUCTION

Three-dimensional electronics (3DE) with high customizability, 3D conformability, and stretchability is becoming an important key factor toward state-of-the-art wearable electronics. This is because of the increasing demands for seamless integration of electronic systems on 3D deformable curvilinear surfaces (e.g., human body–monitoring system, automotive system, and appliances) and for flexible fabrication processes that satisfy the users’ diverse design needs (e.g., biomedical sensor, wearable sensor, and 3D printed sensor) (*1*). While 2D film-based flexible electronics can be deformed and attached onto curved surfaces (*2*–*5*), it is still challenging to conformally attach them onto the complicated and irregular 3D surfaces with both convex and concave shapes with various curvature radii. From this premise, processes for the direct fabrication of 3DE with high customizability, 3D conformability, and stretchability onto any complicated surface are in high demand. Recently, a variety of fabrication methods such as 3D printing (*6*–*9*), laser direct structuring (*10*, *11*), spraying (*12*), hydrographic printing (*13*–*15*), and thermoforming (*16*–*19*) have been used to fabricate 3DE. Among these fabrication methods, thermoforming, a manufacturing method that uses the thermoplastic deformation of a plastic film onto a specific 3D-shaped mold, has ample advantages such as low fabrication cost, large area scalability, and quick prototyping capability.

Previously, Schüller *et al*. (*20*) and Zhang *et al*. (*21*) successfully demonstrated 3D color image transfer, whereby, an originally designed 3D image is distorted into a 2D image and then finally transferred to the target 3D surface through the thermoforming process. After that, Ting *et al*. (*19*) introduced the method of generating 3DE based on thermoforming (referred to as “in-mold electronics”). Although this research showed that thermoforming has a great potential for fabricating 3DE, it is still challenging to apply their technologies to fabricate 3DE with high customizability, 3D conformability, and stretchability. First, the conductive silver ink (ME603, DuPont, USA) that they used as a thermoformable conductor is only stretchable up to 35% of thermoplastic deformation, which prevents its application on circuit pattern areas with local strains of more than 35% during the thermoforming process. Thus, this method has low fabrication degrees of freedom. Furthermore, the fabricated 3DE using this method cannot be applied to wearable electronics because of the nonstretchability of the conductive silver ink and the rigid substrate after the thermoforming process. Second, this method contains several iterative processes and calibration steps, which increase fabrication complexity. For these reasons, appropriate stretchability of the film and the electrode and a thermoforming simulation based on thermomechanical properties are needed to achieve the fabrication of highly customizable, conformal, stretchable 3DE. However, to the best of our knowledge, there has not been any research reporting any 3DE fabrication process that meets these requirements, as shown in table S1.

**Fig. 1.**
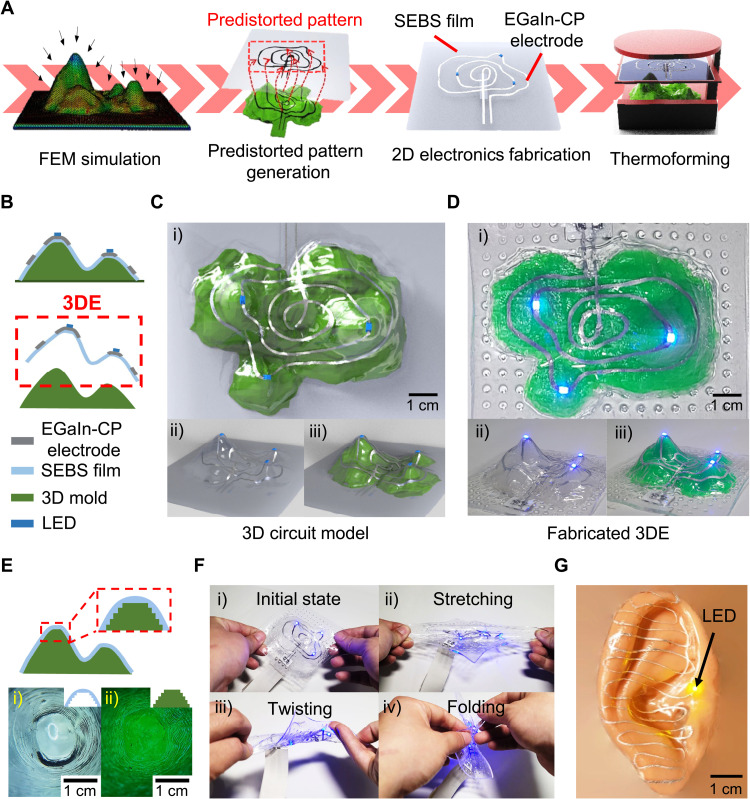
Overview of the fabrication method of 3DE based on predistorted pattern generation and thermoforming process (referred to as PGT3DE) featuring high customizability, conformability, and stretchability. (**A**) Basic mechanism of the PGT3DE. 3DE can be fabricated using thermoforming simulation and the predistorted pattern generation method. (**B**) Schematic cross-sectional illustration of fabricated 3DE based on PGT3DE. (**C** and **D**) Designed 3D circuit model (C) and fabricated 3DE (D) [(i) top view and (ii and iii) bird’s eye view without the 3D mold (ii) and with the 3D mold (iii)]. (**E**) 3D conformal property of the thermoformed SEBS film with a microscope image of thermoformed SEBS film (i) and 3D mold (ii). (**F**) Electrical stability under various deformations; the 3DE is robust under stretching (ii), twisting (iii), and folding (iv) deformations without electrical disconnection. (**G**) Ear-shaped 3DE. The LEDs in the 3DE are well lit because of the successful electrical interconnection. Photo credit: Jungrak Choi, Korea Advanced Institute of Science and Technology (KAIST).

We therefore propose a fabrication method of 3DE based on predistorted pattern generation and the thermoforming process (hereinafter referred to as “PGT3DE”), with a thermoplastic elastomer and a liquid metal–based conductive electrode. To overcome the limitations of the previously developed thermoforming process, we applied a highly stretchable thermoplastic elastomer–based substrate such as styrene-ethylene-butylene-styrene (SEBS) and a stretchable conductive electrode such as eutectic gallium-indium–based liquid metal mixed with copper microparticles (EGaIn-CP) with thermomechanical property–based 3DE fabrication process. Compared to the rigid thermoplastic material such as polyethylene terephthalate glycol used in previous research, SEBS is a soft thermoplastic elastomer with both elastic and plastic properties, which makes thermoforming process more intricate. In detail, the previous process of 3DE design (iterative processes) with the calibration sheet cannot be applied to SEBS-based 3DE fabrication. The deformation of the SEBS film is affected by the attached calibration sheet because the calibration sheet has higher modulus than the SEBS film. On the other hand, we conducted thermomechanical property–based simulation to obtain the desired 3DE, which can be applied to various 3D shapes without the calibration sheet. Furthermore, the EGaIn-CP electrodes do not affect the deformation of the SEBS film and can maintain high conductivity during the thermoforming process. From this, the PGT3DE has high accuracy and high fabrication degrees of freedom for designing and fabricating the 3DEs. The basic mechanism of the PGT3DE is presented in Fig. 1A, and it is summarized as follows: The 3DE can be fabricated using the finite element method (FEM) for thermoforming simulation and a predistorted pattern generation method. For thermoforming FEM simulation, thermomechanical properties of the thermoplastic elastomer film are measured. Therefore, the designed 3DE can be converted into a predistorted 2D pattern using the FEM simulation and then precisely transformed to the desired 3D shape via the thermoforming process. Here, the 3DE has high 3D conformability because the thermoforming process enables complete replication of both the overall shape and the surface texture of the 3D mold compared to other existing fabrication methods for the 3D-shaped electronics such as 3D printing, laser direct structuring, spraying, and hydrographic printing. Furthermore, the PGT3DE allows for high thermoformability during the device fabrication and high stretchability during the device operation owing to the use of a highly stretchable thermoplastic elastomer–based substrate such as SEBS and a stretchable conductive electrode such as EGaIn-CP. Regarding thermoformability and stretchability, SEBS substrate can undergo thermoplastic deformation of up to 100% during the thermoforming process for the device fabrication and also has a large elastic range up to 600% (when stretched) during device operation of the 3DE. In addition, the EGaIn-CP electrode embedded in the SEBS substrate maintains high electrical conductivity during fabrication and operation of the device. Using the PGT3DE, we demonstrated practical applications such as a 3D wearable touch sensor and a speaker with 3D geometries. Furthermore, we applied PGT3DE to demonstrate a wireless battery-free 3D system, which can be combined with sensors, for showing its potential usage in various IoT (internet of things) applications. We envision that the demands for new customizable, 3D conformal, and stretchable 3DE will explode in numerous industrial sectors such as biomedical, robotics, health care, and entertainment industries in the near future. We believe that the PGT3DE can enable a wide range of new functionalities in 3DE and could be an innovative and efficient solution to the design and fabrication of 3DE.

## RESULTS

### Overview of PGT3DE featuring high customizability, 3D conformability, and stretchability

Figure 1 shows an overview of PGT3DE. Structurally, 3D circuit models with arbitrary 3D surfaces can be designed. For example, a 3D electronic circuit with three light-emitting diodes (LEDs) on the mountaintops and their interconnection via EGaIn-CP electrodes is made as presented in Fig. 1C. The 2D schematic cross-sectional illustration of fabricated 3DE based on PGT3DE is shown in Fig. 1B, and the designed 3D circuit model of the 3DE and the 3D mold are shown in Fig. 1C. On the basis of this 3D circuit model, the 3DE is fabricated using a combination of 3D modeling, FEM simulation, and predistorted pattern generation technique (Fig. 1D). Three LEDs on the 3D surface are lit brightly, enabled by the good electrical connection formed between the LEDs and the EGaIn-CP electrodes on the 3D surface. Regarding the 3D conformability of the 3DE, the thermoforming process enables the complete replication of both the overall shape and the surface texture of the 3D mold with both convex and concave shapes with various curvature radii (Fig. 1E and fig. S1). The inner surface of the thermoformed SEBS film has a conformal contact with the 3D mold surface by forming the reverse texture of the mold. Regarding the stretchability of the 3DE, SEBS as a highly stretchable thermoplastic elastomer substrate and EGaIn-CP, as a stretchable electrode material, enable the 3DE to be reliable under stretching, twisting, and folding deformations without electrical disconnection (Fig. 1F and movie S1). Using the PGT3DE, other complicated shapes such as ear-shaped 3DE with many convex and concave topologies can be fabricated as shown in Fig. 1G. In this figure, successful electrical interconnection on the complex 3D surface can be verified by the well-lit LED.

### Fabrication process of PGT3DE

**Fig. 2.**
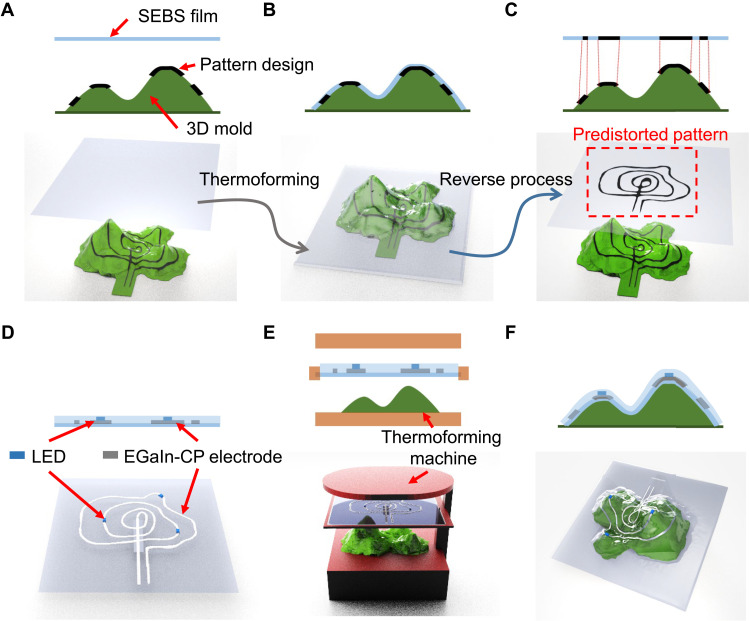
Fabrication process of the PGT3DE. (**A**) Design of 3DE circuit pattern on 3D mold. (**B**) Thermoforming simulation. (**C**) Predistorted pattern generation. (**D**) Patterning the EGaIn-CP electrode and mounting electronic devices on 2D planar SEBS film based on the predistorted pattern. (**E**) Thermoforming process. (**F**) Fabricated 3DE.

The 3DE based on PGT3DE is produced by following the steps illustrated in Fig. 2. Overall, the process is divided into two parts. In the first part (Fig. 2, A to C), the predistorted 2D pattern is generated using an FEM simulation and 3D modeling of the designed 3DE. In the second part (Fig. 2, D to F), EGaIn-CP electrodes and electronic devices (e.g., LEDs) are patterned and placed on the 2D planar SEBS film using the predistorted 2D pattern. Then, the 2D planar SEBS film is heated until it becomes pliable, and then, it is plastically deformed to the designed 3DE during the thermoforming process. As a preliminary step to the first part, a 2D planar mesh with the thermomechanical properties of SEBS and a 3D mold mesh are prepared for the precise FEM simulation. In more detail, a circuit pattern of the 3DE is designed on the 3D mold surface using a 3D modeling tool (Fig. 2A). Next, during the thermoforming simulation, the 2D planar mesh deforms conformably onto the surface of the 3D mold. After that, a mapping between the deformed 3D mesh and the surface of the 3D mold mesh is established, and then, the circuit pattern on the 3D mold is transferred to the deformed 3D mesh (Fig. 2B). Using this one-to-one correspondence between 2D planar mesh and the deformed 3D mesh, the pattern on the deformed 3D mesh is distorted to the 2D planar mesh (Fig. 2C). Here, this distorted pattern on the 2D planar mesh is referred to as the “predistorted pattern.” On the basis of the predistorted pattern, the mask for the stencil printing of the EGaIn-CP electrode is fabricated. Subsequently, the electrode is stencil-printed on the 2D planar SEBS film, and the electronic devices are mounted. After that, the electronics is passivated with a thin SEBS film by spin coating (Fig. 2D). The next step is thermoforming; the 3D mold used during the thermoforming process is fabricated by a digital light processing 3D printer with a heat resistance resin or a silicone casting mold. After that, the 2D planar SEBS film with electronics is placed on a thermoforming machine and heated up to the softening point of SEBS. Then, the film is stretched over the surface of the 3D mold by applying vacuum from beneath the mold (Fig. 2E). Last, the 3DE fabrication is completed by detaching it from the 3D mold (Fig. 2F).

### Thermoforming simulation

**Fig. 3.**
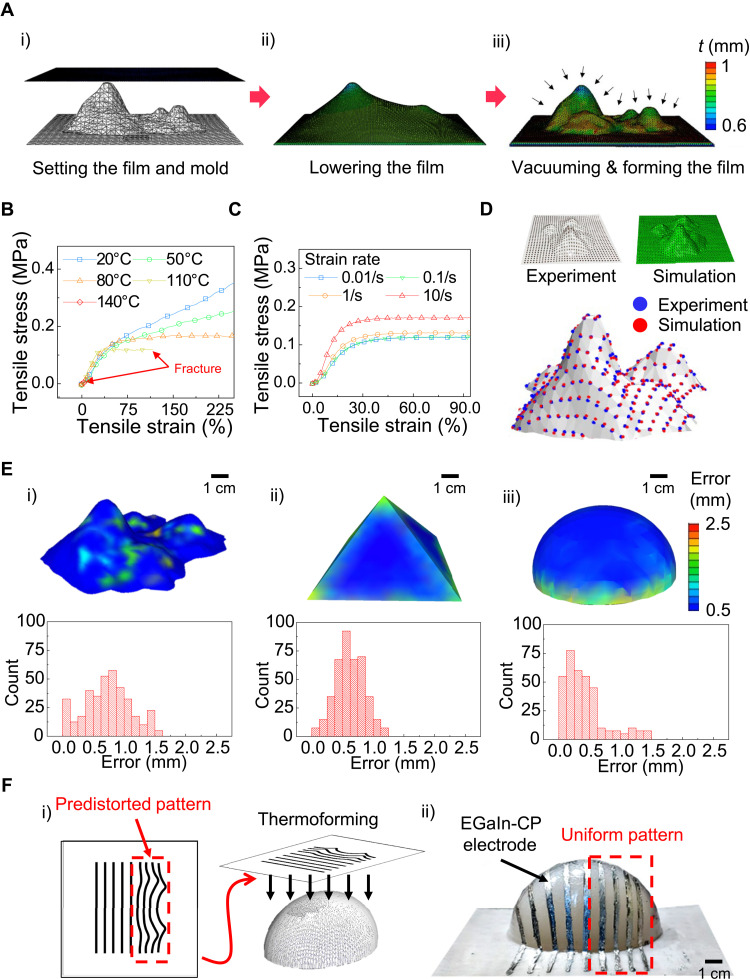
Numerical and experimental analysis of thermoforming process. (**A**) Thermoforming simulation process. (i) The 3D mold mesh is placed on the bottom plate, and the 2D planar mesh is placed above the 3D mold mesh. (ii) The 2D planar mesh is lowered. (iii) Vacuum pressure is applied to the 2D planar mesh that is pulled toward the 3D mold mesh. (**B**) Stress-strain curves of the SEBS film at different temperatures (at a strain rate of 1/s). (**C**) Stress-strain curves of the SEBS film at different strain rates (at a temperature of 110°C). (**D**) Comparison between the simulated results and the experimental results. There is high similarity between the simulation result and the 3D reconstruction of the experimental result. (**E**) Visualization of distance errors between the 3D reconstruction and the simulated results using 3D color map and histograms for various shapes: mountain (i), pyramid (ii), and parabola (iii). (**F**) 3D line patterning of EGaIn-CP electrodes. (i) Predistorted pattern generation and thermoforming. (ii) Fabricated result. On the right side, uniform 3D line patterns with similar dimensions could be obtained with the predistorted pattern compared to pristine patterns on the left side. Photo credit: Jungrak Choi, KAIST.

The thermoforming process involves complex thermoplastic deformation when the 2D planar thermoplastic film adapts to the 3D mold. Thus, the “thermoforming simulation” step described briefly in Fig. 2B will be elaborated more in this section. As previously explained, FEM for thermoforming simulation is used to obtain the predistorted 2D pattern. To simplify the FEM simulation, we made the following assumptions. First, the interaction between the 2D planar mesh with the thermomechanical properties of SEBS and the 3D mold mesh is a hard contact without sliding. Second, the temperature of the 2D planar mesh is uniform. Third, the vacuum pressure is applied instantaneously. The simulation is conducted by following the sequential steps described in Fig. 3A. First, the 3D mold mesh is placed on the bottom plate and the 2D planar mesh is placed above the 3D mold mesh (Fig. 3Ai). Second, the 2D planar mesh is lowered (Fig. 3Aii) and then the vacuum pressure is applied, causing the deformation toward the 3D mold mesh (Fig. 3Aiii). The video of the thermoforming simulation is shown in movie S2. For the precise thermoforming simulation, thermomechanical properties of SEBS should be measured. The 2D planar SEBS film has both thermoplastic and elastic properties that are dependent on the temperature and the strain rate. The glass transition temperature (*T*_g_) of SEBS was estimated, using differential scanning calorimetry (DSC), to be around 70°C (fig. S2). Generally, a thermoplastic elastomer undergoes an elastic deformation at temperatures lower than *T*_g_ (*T* < *T*_g_), and a combination of plastic and elastic deformations at temperatures higher than *T*_g_ (*T* > *T*_g_). In the latter case, plastic deformation becomes more dominant at higher temperature. We found that 110°C is the operation temperature for thermoforming, which can provide the SEBS film with the maximum plastic deformation of 100% without mechanical failure while keeping the geometry after finishing the thermoforming process (fig. S3). As shown in Fig. 3B, at *T* = 110°C and a strain rate of 1/s, the SEBS film undergoes a plastic dominant deformation after the initial yield point, which means that the deformed SEBS will not return to the original shape. As shown in Fig. 3C, the stiffness of the SEBS film is also influenced by the strain rate. Higher strain rate increases the stiffness of the SEBS film. Here, it should be noted that strain rates higher than 10/s could not be applied because of the limitation of our tensile testing machine. Using these thermomechanical tests for the SEBS film, the material properties of the 2D planar mesh in the simulation are set to match the thermomechanical properties of the SEBS film at 110°C. To compare the simulation and experimental results, we obtained a 3D reconstruction of the experimental result by the following method. A periodic dot array pattern with a 3-mm pitch was printed on the 2D planar SEBS film. After thermoforming, a 3D model of the thermoformed SEBS film with distorted dot patterns was reconstructed using photogrammetry (i.e., 3D reconstruction from 2D photos taken at different angles). As shown in Fig. 3D, the simulation and experimental results agree well with a close proximity for each dot. Furthermore, for a quantitative evaluation of various thermoformed SEBS films on different 3D molds such as a mountain, a pyramid, and a parabola, the displacement errors for individual dots (i.e., distance between corresponding dots in the simulation and experimental results) were calculated and presented in Fig. 3E using a 3D color map and a histogram. In the color map, the error ranges between 0 and 2 mm. We obtained an average displacement error of 1.1, 1.2, and 0.7 mm for the mountain, the pyramid, and the parabolic molds, respectively. These errors can be reduced with the use of precise heating and positioning. Furthermore, this thermoforming simulation could be applied to the patterning of uniform lines on a 3D curved surface, in this case, on a parabola-shaped mold (Fig. 3F). To illustrate the enhanced results by the PGT3DE, we predistorted the line pattern on the right side of the 2D planar SEBS film only, while the left side was printed with 2D parallel line patterns (nondistorted line patterns) with equal dimensions; subsequently, the 2D planar SEBS film was thermoformed on the parabola-shaped mold (Fig. 3Fi). As shown in Fig. 3Fii, the line patterns on the left side exhibited unequal dimensions because of nonuniform extensions at different locations. In contrast, on the right side, uniform 3D line patterns with similar dimensions could be obtained with the predistorted patterns; this shows the enhanced results of using PGT3DE. Further quantitative evaluation of 3D line patterns is shown in fig. S4.

### Electrical characteristics of 3DE based on the PGT3DE

**Fig. 4.**
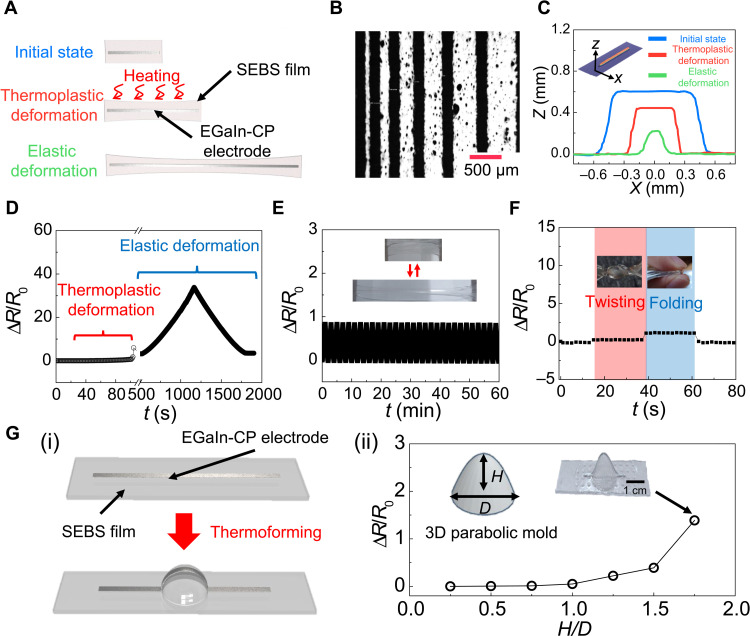
Electrical characteristics of 3DE based on the PGT3DE. (**A**) Schematic illustration of the 3DE with a line pattern of EGaIn-CP electrode during thermoforming process (thermoplastic deformation) and during stretching in the 3DE operation (elastic deformation). (**B**) Microscope image of line-patterned EGaIn-CP electrodes. (**C**) Dimension change of EGaIn-CP electrode during thermoforming and stretching in the 3DE operation. (**D**) Resistance change of the 3DE during thermoforming and stretching in the 3DE operation. (**E**) Repeatability test of the 3DE with 1000 repeated cyclic loadings of 100% tensile strain. (**F**) Resistance change of the 3DE during twisting and folding. (**G**) Resistance change of parabola-shaped 3DE. (i) Schematic illustration of fabrication process. (ii) Resistance change of the parabola-shaped 3DEs after thermoforming using different *H*/*D* values of parabolic molds. Photo credit: Jungrak Choi, KAIST.

An EGaIn-CP electrode has been recently reported as a promising electrode material for stretchable devices due to its high electrical conductivity, adhesive property, and stretchability (*22*–*24*). However, previous studies investigated the usage of EGaIn-CP as a stretchable electrode in elastic deformation only. On the other hand, in the present work, we introduce the new possibility of using EGaIn-CP as a thermoformable electrode material. As shown in fig. S5, the EGaIn-CP gradually solidifies with different CP contents. This change is due to a particle internalization process between CPs and EGaIn in a HCl solution. Using this characteristic, the EGaIn-CP with appropriate CP contents can be used for stencil printing. As shown in fig. S6, 10:1.5 weight ratio of EGaln:CP is the optimal proportion for the stencil printing. Figure 4 shows the electrical characteristics of the 3DE during the thermoforming process (through which it undergoes thermoplastic deformation) and during operation of the 3DE, i.e., while it is stretched (through which it undergoes elastic deformation). The basic electrical properties can be evaluated by measuring the relative resistance change (Δ*R*/*R*_0_) during the tensile tests of the thermoplastic and elastic deformations (Fig. 4A). For the preparation of the tensile test, the EGaIn-CP electrode was line-patterned on a 2D planar SEBS film and covered with a thin SEBS film by spin coating. Regarding the pattern resolution of the EGaIn-CP electrode, two different printing methods were used. Stencil printing was used for the line patterns with widths larger than 500 μm, whereas a combination of stencil printing and fiber laser patterning was used for the patterns with widths of 150 μm (Fig. 4B). From the 3D confocal microscope images in fig. S7, it could be confirmed that the EGaIn-CP electrode has high fluidity, providing an electrical continuity during the thermoplastic and elastic deformations. In addition, from the cross-sectional image in Fig. 4C, it was observed that the cross-sectional area decreases when the length of the EGaIn-CP electrode increases during the thermoplastic and elastic deformations. Therefore, Δ*R*/*R*_0_ of the EGaIn-CP electrode gradually increases without electrical disconnection during the thermoplastic deformation (strain = 100%) and during the elastic deformation (strain = 600%) as presented in Fig. 4D. For the stretchable 3DE device operation, it is important to have excellent signal recovery characteristics to ensure stable performance and a long lifetime. After thermoplastic deformation, the EGaIn-CP electrode showed a long-term stability in response to 1000 repeated cyclic loadings with an elastic deformation of 100% tensile strain (Fig. 4E). The signal drifting and structural changes were not notable during the cyclic test owing to the stable mechanical and electrical properties of the EGaIn-CP electrode and the SEBS film. Furthermore, there were no significant changes in the responses to twisting and folding of the fabricated 3DE (Fig. 4F). To evaluate the dependence of electrical characteristics on dimensions of 3D mold and temperature, we thermoformed the line-patterned EGaIn-CP electrode on the 2D planar SEBS film onto parabolic molds (Fig. 4G). Then, Δ*R*/*R*_0_ of different 3D parabola-shaped 3DEs was measured. Specifically, the parabolic molds were 3D printed with a dimeter (*D*) of 20 mm and different heights (*H*). Here, the aspect ratio of the parabolic mold is defined as *H*/*D*. As shown in Fig. 4Gii, Δ*R*/*R*_0_ of the 3DE gradually increases with increasing *H*/*D* values because higher values of *H*/*D* induce more local stresses. Notably, when the value of *H*/*D* was higher than 1.75, the EGaIn-CP electrode was disconnected because of mechanical failure of the SEBS film. However, *H*/*D* is lower than 1.75 in most 3D shapes, and thus, the electrical disconnection of EGaIn-CP does not occur during the thermoforming process. Given that we used SEBS as the substrate for the 3DE, the 3DE is inherently affected by the temperature. Therefore, Δ*R*/*R*_0_ of the parabola-shaped 3DE with *H*/*D* of 1 under different temperature conditions was measured to verify that the 3DE can withstand while keeping its structure without electrical disconnection. For the test, the 3DE was put into a convection oven under different temperature conditions for 10 min. After that, the 3DE was cooled down, and Δ*R*/*R*_0_ of the 3DE was measured. As shown in fig. S8, the 3DE kept its original shape and resistance up to 65°C. When the temperature was higher than 65°C, the shape of the 3DE collapsed, which caused a marked increase of Δ*R*/*R*_0_ of the EGaIn-CP electrode and electrical disconnection of the EGaIn-CP electrode. Therefore, the 3DE can be operated up to the temperature of 65°C.

### Demonstration of 3DE for sensor and actuator applications

**Fig. 5.**
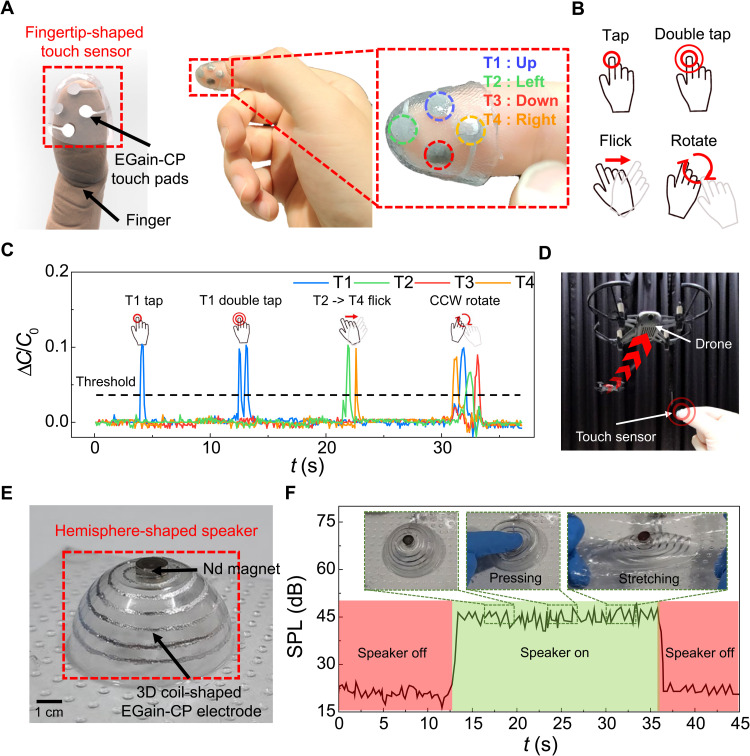
Demonstration of 3DE for touch sensor and speaker applications. (**A** to **D**) Fingertip-shaped capacitive touch sensor. (A) Schematic illustration and photographs of the fingertip-shaped touch sensor. The sensor is composed of four touch pads (T1, T2, T3, and T4). (B) Gesture control functions. Four touch pads allow not only four simple taps but also several gesture controls such as double tap, flick, and rotation. (C) Capacitance changes over time when gesture control functions were applied. CCW, counterclockwise. (D) Quadcopter drone control using the touch sensor. The touch sensor was successful in controlling a quadcopter drone. (**E** and **F**) Hemisphere-shaped speaker. (E) Photograph of the hemisphere-shaped speaker. (F) Characteristic of the speaker. The speaker generated sound well with little sound distortion under mechanical deformations such as pressing and stretching. Photo credit: Jungrak Choi, KAIST.

We present potential applications of the proposed technology by demonstrating a 3D wearable touch sensor and a speaker with 3D geometries (Fig. 5). First, for the sensor application, a fingertip-shaped capacitive touch sensor was demonstrated as shown in Fig. 5 (A to D). Because the fingertips are one of the most sensitive body parts, precise positioning of touch pads and stretchability of a sensor are essential for the user’s comfort. Although some fingertip-type sensors already exist, they have been limited to rigid sensors mounted on a rigid frame, which hardly conform to the shape of the fingertip. Therefore, a fingertip-type sensor with a soft frame has been developed (*25*, *26*). However, it is still challenging to place electronic components on highly curved surfaces with high accuracy. Our fingertip-shaped touch sensor was made of EGaIn-CP–based touch pads mounted on the fingertip-shaped SEBS substrate. Thus, users can wear the sensor comfortably and press the touch pad while the sensor maintains stable electrical connection. The thermoforming process and the precise thermoforming simulation–based patterning explained in this report enabled fast and precise fabrication of the touch sensor. The sensor is composed of four touch pads that allow not only four simple taps but also several gesture controls such as double tap, flick, and rotation; some gestures make use of multiple sensing touch pads (Fig. 5B and movie S3). Gesture control functions are realized through time-based grouping of the signal (Fig. 5C). When successive signals are detected within a certain time window, the signals are interpreted as a one-group signal, instead of multiple individual signals. Furthermore, to avoid false detections, the signal must exceed a certain threshold. Therefore, the touch sensor was successful in controlling a quadcopter drone (Fig. 5D and movie S4). The drone was controlled with various gesture control functions to achieve different movements such as landing, taking off, forward/backward/left/right movement, flip, and rotation.

Second, for the actuator application, a 3D-shaped speaker was demonstrated, as shown in Fig. 5 (E and F). Previously, a 2D stretchable speaker based on the electromagnetic interaction between a liquid metal coil and a neodymium (Nd) magnet was reported as a body-attached acoustic device (*27*). However, because a human body is composed of various 3D curvatures, the 2D stretchable speaker cannot be conformally attached onto the 3D surface of the human body such as an elbow, a shoulder, and a heel. Therefore, we demonstrated a hemisphere-shaped speaker for showing the potential of the 3D stretchable speaker. The hemisphere-shaped speaker was fabricated by embedding the Nd magnet in the 3DE, as shown in Fig. 5E. To verify the sound generation, we applied an audio frequency electrical signal to the 3D-shaped speaker. Then, the sound was measured using a microphone that was positioned 10 cm above the center of the 3D-shaped speaker. While the music was played, the sound was successfully generated from the 3D-shaped speaker with little sound distortion (movie S5). As presented in Fig. 5F, successful operation of the 3D-shaped speaker could be clearly observed by measuring the sound pressure level (SPL). In addition, SPL was not affected by mechanical deformations of the speaker such as pressing and stretching.

### Demonstration of 3DE for wireless battery-free system

**Fig. 6.**
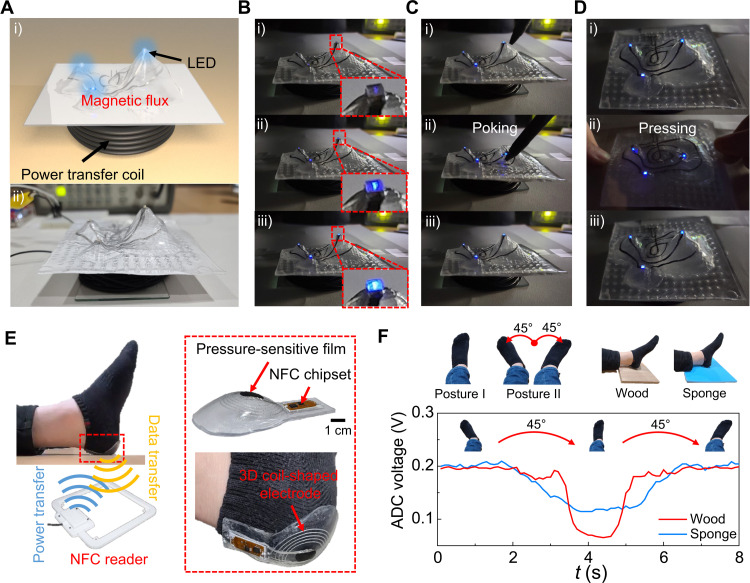
Demonstration of 3DE for wireless battery-free system with a near-field communication. (**A** to **D**) Wireless power transmission system using the mountain-shaped 3DE. (A) Schematic illustration and photograph of the wireless power system. (B) Intensity change of the LEDs. The intensity of the LEDs gradually increased with increase in induced current. (C and D) Negligible change in the light intensity of the LEDs while poking with a sharp object (C) or pressing with a transparent rigid film (D). (**E** and **F**) Heel-shaped wireless battery-free pressure sensor for bedsore monitoring system with NFC. (E) Schematic illustration and photographs of the heel-shaped pressure sensor. (F) Pressure sensor signal from the NFC reader during various dynamic foot motions. The pressure signal was measured well from NFC reader and alternated between the maximum pressure at posture I and the minimum pressure at posture II as the angle of the foot changed. Photo credit: Jungrak Choi, KAIST.

Wireless technologies can realize the connection of remote electronic devices such as wireless sensors for IoT applications. Therefore, we applied the PGT3DE to demonstrate wireless battery-free 3DE systems (Fig. 6). First, a 3D-shaped wireless power transmission system was developed using mountain-shaped 3DE, as previously introduced in Fig. 1. The mountain-shaped 3DE is composed of three LEDs on mountaintops and their interconnections via a 3D coil-shaped EGain-CP electrode, which can receive wireless power to illuminate the LEDs. To operate the wireless power transmission system, we used a simple transistor oscillator circuit that generates a radio frequency power. The transistor in the circuit opens and closes the circuit at a frequency of 70 kHz, thus producing quick bursts of current into the power transfer coil. These changes in the current inside the power transfer coil produce a magnetic field, which then induces an electrical current inside the electrode in the 3DE and, consequently, the lighting of the LEDs. When the 3DE is centered on top of the power transfer coil, the LEDs are lit well (Fig. 6A). After that, different amounts of current were applied to the oscillator circuit (Fig. 6B). The intensity of the LEDs gradually increased, while the induced current into the oscillator circuit was raised. Furthermore, because of the usages of SEBS and EGaIn-CP as stretchable materials, negligible change in the light intensity of the LEDs was observed while poking the device with a sharp object or while pressing with a transparent rigid film (Fig. 6, C and D, and movie S6). It can also be confirmed that the geometry of the 3DE returned to the initial geometry well after poking and pressing. Furthermore, another 3D-shaped wireless power transmission system is also demonstrated in fig. S9 and movie S7.

After verifying the wireless power transmission to the 3DE with the 3D coil-shaped electrode, we further developed a 3D-shaped battery-free pressure sensor with near-field communication (NFC). This device is composed of a pressure-sensitive film, a 3D coil-shaped electrode, and an NFC chipset to establish wireless power and transmit pressure sensor data to an NFC reader. As a feasibility test, a hemisphere-shaped pressure sensor was fabricated and demonstrated in fig. S10 and movie S8. After that, a heel-shaped wireless battery-free pressure sensor for a bedsore monitoring system was developed (Fig. 6, E and F). The 3DE is well attached to the heel because of the customized design of the 3DE based on the geometry of the heel (Fig. 6E). From the NFC reader, continuous pressure measurements could be acquired successfully. In evaluating the bedsore monitoring system, the pressure was measured under different lying positions: posture І (toe pointing an angle of 90° to a floor) and posture ІІ (toe pointing an angle of 45° to a floor). This experiment was done using materials of different hardness for the floor, namely, a wood board and a soft sponge. As shown in Fig. 6F and movie S9, the pressure signal was measured well from the NFC reader during various dynamic motions of the foot between postures І and ІІ. As the angle of the foot changed, the pressure alternated between the maximum pressure at posture I and minimum pressure at posture II because the pressure sensor of the 3DE was designed to measure a normal pressure at posture I. Furthermore, when the foot was lying on the wood board, a higher maximum pressure was observed as compared to when it was lying on the soft sponge, because the soft sponge deconcentrates the applied pressure at the foot-floor interface.

## DISCUSSION

This paper reports a manufacturing method (PTG3DE) to fabricate 3DE based on predistorted pattern generation and the thermoforming process. On the basis of the precise thermoforming simulation and highly stretchable materials such as SEBS and EGaIn-CP–based electrodes, the PTG3DE enables to fabricate the highly customizable, conformal, stretchable 3DE. Hence, this method enables customizable design and 3D conformal contact to various complicated target surfaces. On the basis of the FEM simulation, the 3DE can be precisely designed and fabricated using predistorted pattern generation based on the user design needs. The use of SEBS- and EGaIn-CP–based electrodes enables the fabricated 3DE to have high stretchability, the inner surface to be conformal to the target surface, and seamless electronics systems. To show the potential usages of the PTG3DE in various industries, we demonstrated practical applications such as a touch sensor, a speaker, and wireless battery-free systems with 3D geometries.

Here, we focused on introducing the core technology of fabricating 3DE with high stretchability and customizability based on the PTG3DE. With further optimization, the PTG3DE reported here has the potential to be implemented as a simple, fast, low-cost, and scalable manufacturing process of 3DE. The following are viable aspects/components to aid in such enhancement: optimization of the thermoformable substrate, electrode, and functional sensing material; thermoforming equipment with higher temperature and position controllability; studies of the thermoforming simulation variability; and implementation of functionalities such as interconnection between liquid metal–based electrode and a commercial integrated circuit (IC) chip. We expect that personalized and customized electronics, with a wider range of wearable electronics functionalities, will be exploded in the near future. We believe that the proposed method will provide a significant contribution to the development of the 3DE with higher functionalities.

## MATERIALS AND METHODS

### Materials

SEBS polymer pellet (HP-10AN) was purchased from Super Asia Polyblend. EgaIn [75 weight % (wt%) of Ga and 25 wt% of In] was purchased from Dongguan Wochang Metal Products. CP (1 μm) was purchased from HKK Solutions. Toluene (99.5%) was purchased from JFACTORYKOREA. Hydrochloric acid solution (10%) was purchased from HangYangsangsa. Thermoforming machine was purchased from Tamsun and customized.

### SEBS film preparation

A pneumatic press machine was used to fabricate a 1-mm-thick SEBS film with a loading pressure of 1 MPa and a temperature of 160°C. To fabricate the 1-mm-thick SEBS, three aluminum plates (square perforated bottom and top plates) were prepared. The SEBS pellet was put into the square perforated plate, and it was placed between the bottom and top plates. Then, the plates were put into the pneumatic press machine, and pressure was applied to the plates for 5 min. After that, the plates were cooled down, and the SEBS film was fabricated by detaching from the plates (fig. S11). For a passivation layer of a thin SEBS film, an SEBS solution, with a SEBS:toluene weight ratio of 1:5, is used for the spin-coating process.

### EGaIn-CP preparation

An EGaIn-CP was fabricated by mixing the EGaIn with the CP. To mix them well, the mixing process was performed in a HCl solution, which can remove the oxide layer on the EGaIn and CP and enable particle internalization. The EGaIn-CP with EGaln:CP weight ratios of 10:0, 10:0.5, 10:1, 10:1.5, 10:2, 10:2.5, and 10:3 were prepared and put into the HCl solution. Then, the two components were sonicated together for 10 min. The EGaIn-CP was obtained after vacuum drying at 60° to 80°C for 3 hours to remove residues of the HCl solution.

### Finite element analysis

Finite element analysis (FEA) was conducted to estimate the mechanical behavior of thermoforming using Abaqus software (Dassault Systemes Simulia Corporation, Johnston, RI). The modeling study focused on the prediction of where and how each mesh point of the planar SEBS film moves onto the surface of the target object.

### Predistorted pattern generation

To generate the predistorted pattern, we used a 3D mesh processing software (MeshLab, Visual Computing Lab, Italy) and a numeric computing software (MATLAB, MathWorks, USA). From the FEA thermoforming simulation, a simulated mesh of the planar SEBS film was obtained. After that, using the 3D mesh processing software, the designed electronic pattern on the 3D model was projected onto the simulated mesh. From the one-to-one correspondence between the initial mesh and the simulated mesh, the predistorted pattern was obtained (fig. S12).

### Quantitative evaluation of the PTG3DE

The thermoformed SEBS film using different 3D molds was 3D reconstructed using photogrammetry to obtain the positions of each dot. After that, the distance between corresponding dots in the simulation and in the experimental results was calculated and visualized using the 3D mesh processing software and the numeric computing software (figs. S13 and S14).

### Characterization of SEBS and EGaIn-CP electrode

Continuous pressure was applied under different temperature conditions using a universal testing machine (Instron 5969, Instron, USA) for full loading-unloading cycles of the SEBS film. To measure the *T*_g_ temperature of SEBS, a DSC (Q90, TA Instruments Inc., USA) was used. The tensile strain was applied in a customized linear stage. The electrical characteristics of the EGaIn-CP electrode was examined using a source meter (Keithley 2400, Tektronix, USA) with Kelvin (four-wire) resistance measurement. The morphology of the EGaIn-CP electrode channel was captured using a confocal laser microscope (VK-X1050, Keyence, Japan).

### Demonstration of fingertip-shaped capacitive touch sensor

The capacitance of each touch pad was measured using a NE555 timer IC. It outputs a square wave signal with a frequency, which is converted to capacitance. Then, a commercial microcontroller board (ESAP8266, Espressif Systems, China) measured the frequency and controlled the quadcopter drone (Tello, DJI, China) (fig. S15). This experiment was approved by the institutional review board (IRB) of Korea Advanced Institute of Science and Technology (KAIST) (IRB no. KH2021-116).

### Demonstration of 3D-shaped speaker

The sound from the 3D-shaped speaker was generated using an audio amplifier board (TPA3118D, Texas Instruments) connected a commercial music player. While the music was played, the sound generated from the 3D-shaped speaker was recorded by using a commercial microphone, which was located 10 cm away from the center of the 3D-shaped speaker (fig. S16).

### Wireless system measurement

For the wireless power transmission system, a function generator (33210A, Keysight Technologies, USA) and a transistor (BD139) were used to operate high alternating current (AC) signal. The function generator generated AC signals with a resonant frequency, and the transistor amplified the signal to the power transfer coil (fig. S17). This produces a magnetic field, which then induces an electrical current inside the electrode in the 3DE and the lighting of the LEDs. For the battery-free pressure sensor for a bedsore monitoring system with NFC, a pressure-sensitive film (Velostat, Adafruit, USA), an NFC chip (RF430FRL15xH NFC, Texas Instruments, USA), and an NFC reader [ISO15693 (ID ISC. LRM2500-A), FEIG, USA] were used. This experiment was approved by the institutional review board of KAIST (IRB no. KH2021-116). The pressure-sensitive film and the 3D coil-shaped electrode were connected to the NFC chip, which was programmed to send the pressure data to the NFC reader. When the NFC chip is close to the NFC reader, the NFC reader reads the data, which are plotted via a computer (fig. S18).

## References

[1] Y. Huang, H. Wu, L. Xiao, Y. Duan, H. Zhu, J. Bian, D. Ye, Z. Yin, Assembly and applications of 3D conformal electronics on curvilinear surfaces. Mater. Horiz. 6, 642–683 (2019).

[2] J. W. Jeong, W. H. Yeo, A. Akhtar, Y. J. Kwack, S. Li, S. Y. Jung, Y. Su, W. Lee, J. Xia, H. Cheng, Y. Huang, W. S. Choi, T. Bretl, J. A. Rogers, Materials and optimized designs for human-machine interfaces via epidermal electronics. Adv. Mater. 25, 6839–6846 (2013). 24327417 10.1002/adma.201301921

[3] D. H. Kim, J. H. Ahn, W. M. Choi, H. S. Kim, T. H. Kim, J. Song, Y. Y. Huang, Z. Liu, C. Lu, J. A. Rogers, Stretchable and foldable silicon integrated circuits. Science 320, 507–511 (2008). 18369106 10.1126/science.1154367

[4] C. Pang, J. H. Koo, A. Nguyen, J. M. Caves, M. G. Kim, A. Chortos, K. Kim, P. J. Wang, J. B. Tok, Z. Bao, Highly skin-conformal microhairy sensor for pulse signal amplification. Adv. Mater. 27, 634–640 (2015). 25358966 10.1002/adma.201403807

[5] K. Sim, S. Chen, Z. Li, Z. Rao, J. Liu, Y. Lu, S. Jang, F. Ershad, J. Chen, J. Xiao, C. Yu, Three-dimensional curvy electronics created using conformal additive stamp printing. Nat. Electron. 2, 471–479 (2019).

[6] G. Loke, R. Yuan, M. Rein, T. Khudiyev, Y. Jain, J. Joannopoulos, Y. Fink, Structured multimaterial filaments for 3D printing of optoelectronics. Nat. Commun. 10, 4010 (2019). 31488825 10.1038/s41467-019-11986-0 PMC6728390

[7] J. Adams, E. B. Duoss, T. F. Malkowski, M. J. Motala, B. Y. Ahn, R. G. Nuzzo, J. T. Bernhard, J. A. Lewis, Conformal printing of electrically small antennas on three-dimensional surfaces. Adv. Mater. 23, 1335–1340 (2011). 21400592 10.1002/adma.201003734

[8] Y. Jo, J. Y. Kim, S. Jung, B. Y. Ahn, J. A. Lewis, Y. Choi, S. Jeong, 3D polymer objects with electronic components interconnected via conformally printed electrodes. Nanoscale 9, 14798–14803 (2017). 28956046 10.1039/c7nr04111j

[9] A. D. Valentine, T. A. Busbee, J. W. Boley, J. R. Raney, A. Chortos, A. Kotikian, J. D. Berrigan, M. F. Durstock, J. A. Lewis, Hybrid 3D printing of soft electronics. Adv. Mater. 29, 1703817 (2017). 10.1002/adma.201703817 28875572

[10] S. Yoon, K. Choi, S. Baek, H. Chang, Electronic circuit patterning on curved surface by direct laser structuring. Int. Conf. Electr. Mach. Syst. 2011, 1–3 (2011).

[11] R. C. Auyeung, M. W. Nurnberger, D. J. Wendland, A. Pique, C. B. Arnold, A. R. Abbott, L. C. Schuette, Laser fabrication of GPS conformal antennas. Photon Process. Microelectron. Photon. III 5339, 292–297 (2004).

[12] F. Cai, S. Pavlidis, J. Papapolymerou, Y. H. Chang, K. Wang, C. Zhang, B. Wang, Aerosol jet printing for 3-D multilayer passive microwave circuitry. Eur. Microwave Conf. 2014, 512–515 (2014).

[13] G. Saada, M. Layani, A. Chernevousky, S. Magdassi, Hydroprinting conductive patterns onto 3D structures. Adv. Mater. Technol. 2, 1600289 (2017).

[14] M. Tavakoli, M. H. Malakooti, H. Paisana, Y. Ohm, D. G. Marques, P. A. Lopes, A. P. Piedade, A. T. Almeida, C. Majidi, EGaIn-assisted room-temperature sintering of silver nanoparticles for stretchable, inkjet-printed, thin-film electronics. Adv. Mater. 30, 1801852 (2018). 10.1002/adma.201801852 29845674

[15] P. A. Lopes, H. Paisana, A. T. Almeida, C. Majidi, M. Tavakoli, Hydroprinted electronics: Ultrathin stretchable Ag–In–Ga E-skin for bioelectronics and human–machine interaction. ACS Appl. Mater. Interfaces 10, 38760–38768 (2018). 30338978 10.1021/acsami.8b13257

[16] K. Wu, Q. Zhou, H. Zou, K. Leng, Y. Zeng, Z. Wu, High precision thermoforming 3d-conformable electronics with a phase-changing adhesion interlayer. Micromachines 10, 160 (2019). 30813578 10.3390/mi10030160 PMC6471248

[17] Y. Yang, T. Vervust, S. Dunphy, S. V. Put, B. Vandecasteele, K. Dhaenens, L. Degrendele, L. Mader, L. D. Vriese, T. Martens, M. Kaufmann, T. Sekitani, J. Vanfleteren, 3D multifunctional composites based on large-area stretchable circuit with thermoforming technology. Adv. Electron. Mater. 4, 1800071 (2018).

[18] S. Y. Lee, S. H. Jang, H. K. Lee, J. S. Kim, S. K. Lee, H. J. Song, J. W. Jung, E. S. Yoo, J. Choi, The development and investigation of highly stretchable conductive inks for 3-dimensional printed in-mold electronics. Org. Electron. 85, 105881 (2020).

[19] J. Ting, Y. Zhang, S. H. Yoon, J. D. Holbery, S. Ma, iMold: Enabling interactive design optimization for in-mold electronics, in *Extended Abstracts of the 2020 CHI Conference on Human Factors in Computing Systems* (2020), pp. 1–7.

[20] C. Schüller, D. Panozzo, A. Grundhöfer, H. Zimmer, E. Sorkine, O. S. Hornung, Computational thermoforming. ACM Trans. Graphics 35, 1–9 (2016).

[21] Y. Zhang, Y. Tong, K. Zhou, Coloring 3D printed surfaces by thermoforming. IEEE Trans. Vis. Comput. Graph. 23, 1924–1935 (2016). 10.1109/TVCG.2016.2598570 28678700

[22] J. Tang, X. Zhao, J. Li, R. Guo, Y. Zhou, J. Liu, Gallium-based liquid metal amalgams: transitional-state metallic mixtures (TransM2ixes) with enhanced and tunable electrical, thermal, and mechanical properties. ACS Appl. Mater. Interfaces 9, 35977–35987 (2017). 28948776 10.1021/acsami.7b10256

[23] R. Guo, H. Wang, X. Sun, S. Yao, H. Chang, H. Wang, J. Liu, Y. Zhang, Semiliquid metal enabled highly conductive wearable electronics for smart fabrics. ACS Appl. Mater. Interfaces 11, 30019–30027 (2019). 31342753 10.1021/acsami.9b08067

[24] J. Yang, W. Cheng, K. Kalantar-Zadeh, Electronic skins based on liquid metals. Proc. IEEE 107, 2168–2184 (2019).

[25] M. Ying, A. P. Bonifas, N. Lu, Y. Su, R. Li, H. Cheng, A. Ameen, Y. Huang, J. A. Rogers, Silicon nanomembranes for fingertip electronics. Nanotechnology 23, 344004 (2012). 22885907 10.1088/0957-4484/23/34/344004

[26] Y. Gao, H. Ota, E. W. Schaler, K. Chen, A. Zhao, W. Gao, H. M. Fahad, Y. Leng, A. Zheng, F. Xiong, C. Zhang, L. C. Tai, P. Zhao, R. S. Fearing, A. Javey, Wearable microfluidic diaphragm pressure sensor for health and tactile touch monitoring. Adv. Mater. 29, 1701985 (2017). 10.1002/adma.201701985 28833673

[27] S. W. Jin, J. Park, S. Y. Hong, H. Park, Y. R. Jeong, J. Park, S. S. Lee, J. S. Ha, Stretchable loudspeaker using liquid metal microchannel. Sci. Rep. 5, 11695 (2015). 26181209 10.1038/srep11695 PMC4504143

