# Supplementary material for: Customizable, conformal, and stretchable 3D electronics via predistorted pattern generation and thermoforming
Source: Sci Adv. 2021 Oct 13;7(42):eabj0694. doi: 10.1126/sciadv.abj0694 (PMC8514101; doi:10.1126/sciadv.abj0694)
Supplement: Supplementary file 1 — Figs. S1 to S18 Table S1 Legends for movies S1 to S9 [file sciadv.abj0694_sm.pdf]

Science Advances
AAAS

Supplementary Materials for

Customizable, conformal, and stretchable 3D electronics via predistorted pattern generation and thermoforming

Jungrak Choi, Chankyu Han, Seokjoo Cho, Kyuyoung Kim, Junseong Ahn, Dionisio Del Orbe, Incheol Cho, Zhi-Jun Zhao, Yong Suk Oh, Hyunsoo Hong, Seong Su Kim, Inkyu Park*

*Corresponding author. Email: inkyu@kaist.ac.kr

Published 13 October 2021, *Sci. Adv.* **7**, eabj0694 (2021)
DOI: 10.1126/sciadv.abj0694

The PDF file includes:

Figs. S1 to S18
Table S1
Legends for movies S1 to S9

Other Supplementary Material for this manuscript includes the following:

Movies S1 to S9

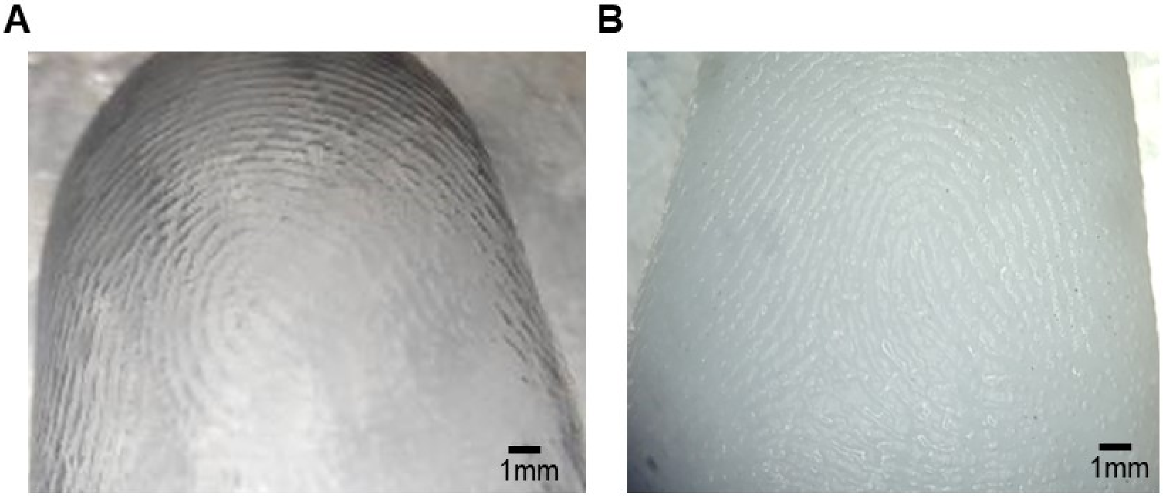

Fig. S1. Surface texture of fingertip-shaped three-dimensional (3D) mold and fingertip-shaped thermoformed styrene-ethylene-butylene-styrene (SEBS) film. The inner surface of the fingertip-shaped thermoformed SEBS film (**A**) has a conformal contact with the 3D mold surface (**B**). Photo Credit: Jungrak Choi, KAIST.

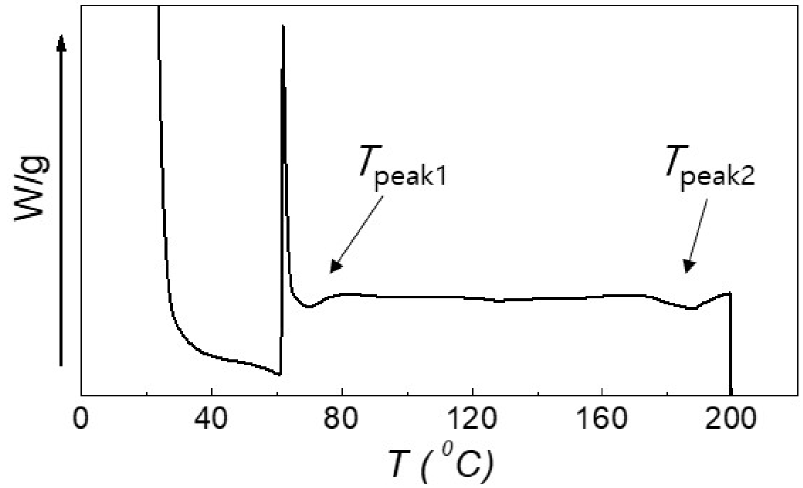

Fig. S2. Differential scanning calorimetry (DSC) curves of SEBS. The glass transition temperature (T_g) and melting temperature (T_m) of SEBS was estimated as around 70 °C and 180 °C using DSC.

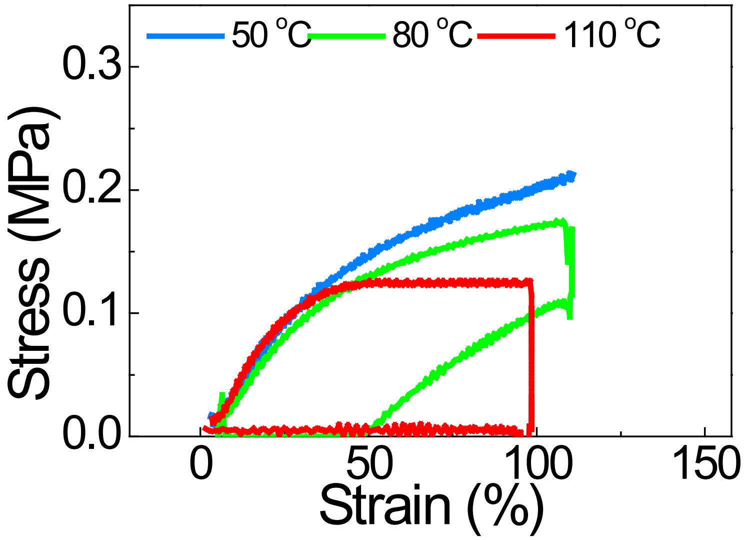

Fig. S3. Stress-strain curves of SEBS film during thermoforming process at different temperatures (at a strain rate of 1 /s). 110 °C is the operation temperature for thermoforming, which can provide the SEBS film with the maximum plastic deformation of 100 % without mechanical failure and keep the geometry after finishing the thermoforming process.

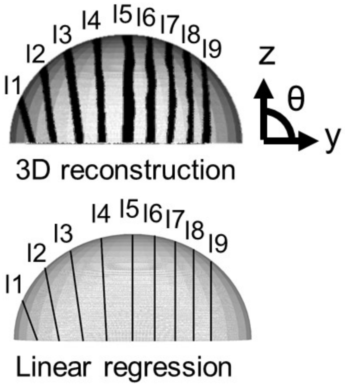

- Angle of inclination

	Non-distorted pattern				Pre-distorted pattern			
	l1	l2	l3	l4	l5	l6	l7	l8	l9
Θ(°)	108	99	98	94	90	90	90	90	90

- Distance

	l1-l2	l2-l3	l3-l4	l4-l5	l5-l6	l6-l7	l7-l8	l8-l9
D (mm)	N/A	N/A	N/A	N/A	11.1	10.7	10.4	10.1

Fig. S4. Calculations of the angles of inclination and the distances from the 3D reconstruction and linear regression of the result. In case of non-distorted pattern, the angle of inclination of the line deviates from 90º when it goes to the end of the 3D hemisphere. Furthermore, due to the non-parallel lines, the distances cannot be calculated. In contrast, in case of pre-distorted pattern, each line is parallel to each other and the distances between two adjacent lines are similar.

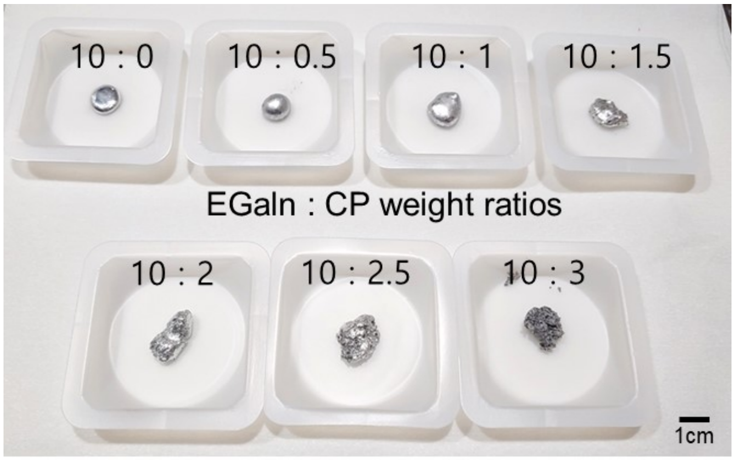

Fig. S5. Eutectic gallium-indium based liquid metal mixed with copper micro particles (EGaIn-CP) with different CP contents. Photo Credit: Jungrak Choi, KAIST.

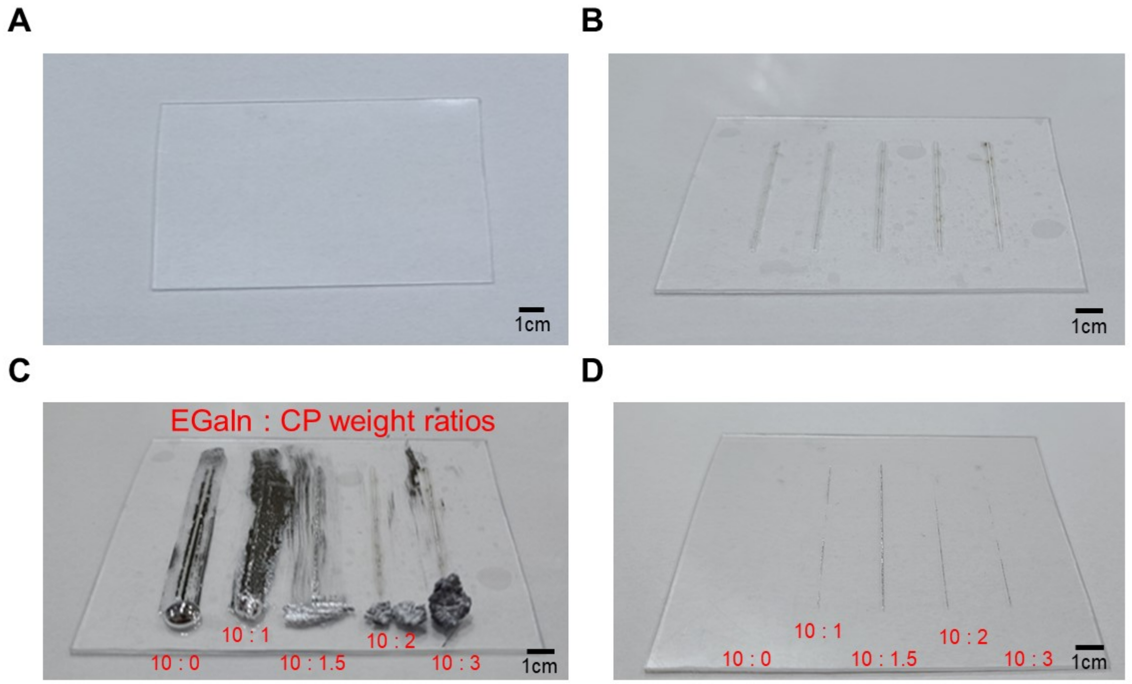

Fig. S6. Stencil printing results of EGaIn-CP electrode. (**A**) SEBS film. (**B**) Stencil printing mask on the SEBS film. (**C**) Stencil printing of EGaIn-CP electrodes with different CP contents. (**D**) Result of stencil printing. 10 : 1.5 weight ratio of EGaln : CP is the optimal proportion for the stencil printing. Photo Credit: Jungrak Choi, KAIST.

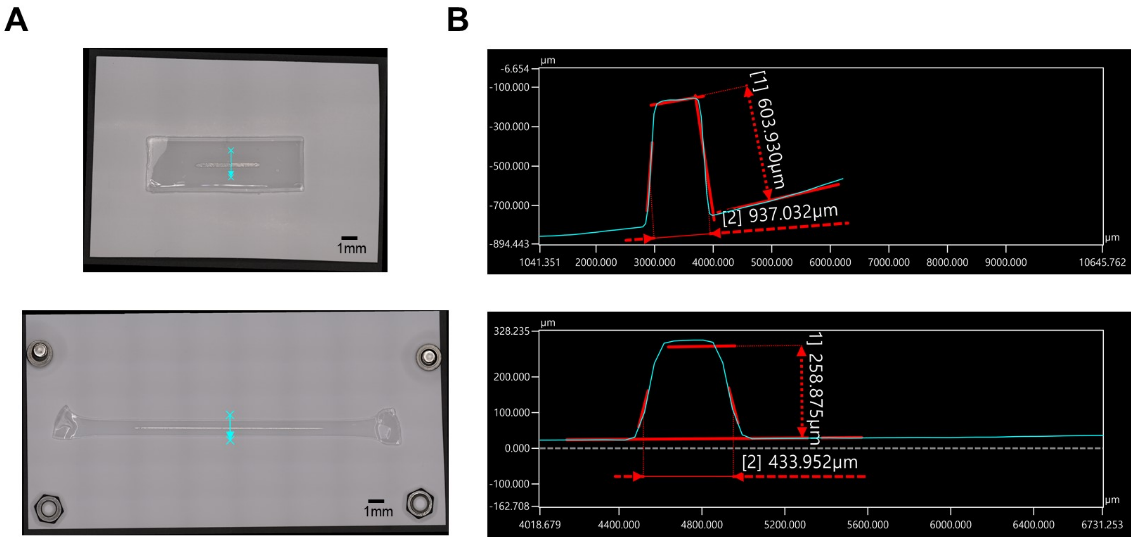

Fig. S7. 3D confocal microscope images of EGaIn-CP electrode. (**A**) Photographs of EGaIn-CP electrode in SEBS film. (**B**) Analysis of channel width and height of EGaIn-CP electrode. It was observed that the cross-sectional area decreases when the length of the EGaIn-CP electrode increases. Photo Credit: Jungrak Choi, KAIST.

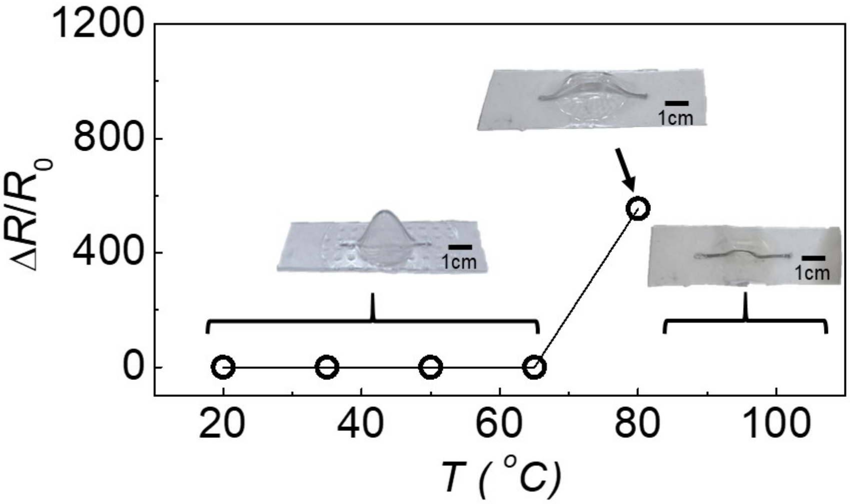

Fig. S8. Resistance change of the parabola-shaped 3DE with H/D of 1 under different temperatures. The 3DE kept its original shape and resistance up to 65 °C. When temperature was higher than 65 °C, the shape of the 3DE collapsed, which caused a dramatic increase of $\Delta R/R_0$ of the EGaIn-CP electrode and electrical disconnection of the EGaIn-CP electrode. Therefore, the 3DE can be operated up to temperature of 65 °C. Photo Credit: Jungrak Choi, KAIST.

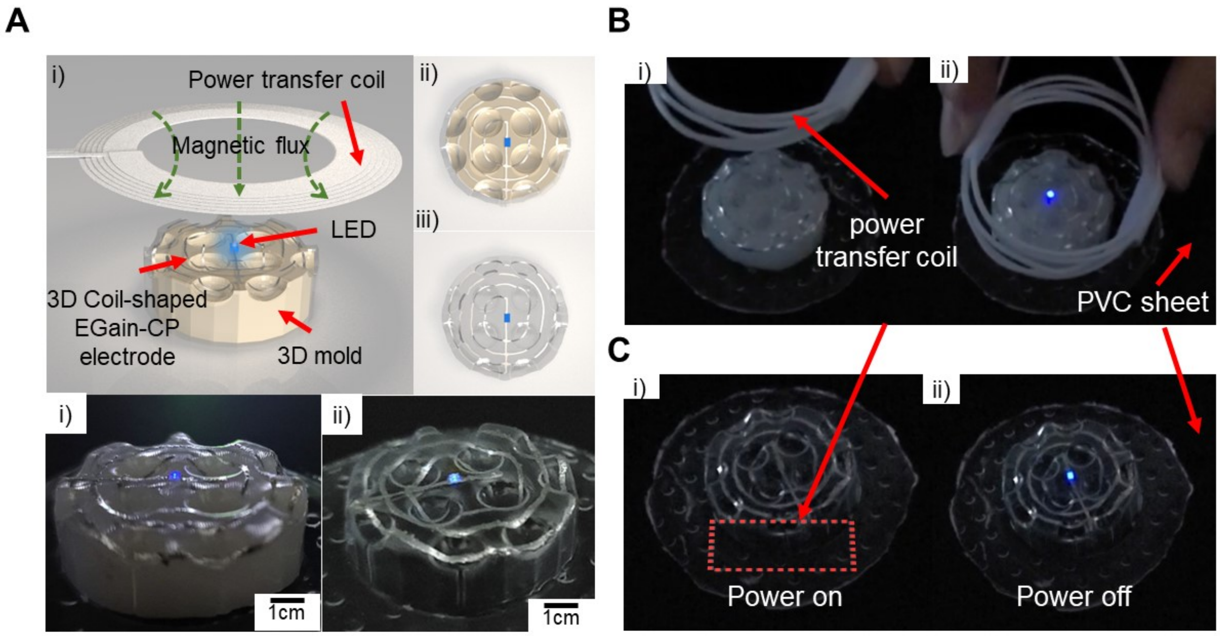

Fig. S9. 3D mogul-shaped wireless power transmission system. (**A**) Schematic illustration and photograph of the wireless power system. (**B**, **C**) Demonstration of the wireless power system when the power transfer coil is placed above the mogul-shaped 3DE. (**B**) Power transfer coil is placed above the mogul-shaped wireless powered LED. When the power transfer coil is close to the mogul-shaped wireless powered LED, the LED is lit. (**C**) Power transfer coil is placed beneath the PVC sheet. Mogul-shaped 3DE can receive the power from the power transfer coil through a PVC (polyvinyl chloride) sheet, as an intermediate layer, because the electromagnetic flux can penetrate some materials such as plastics and glasses. Photo Credit: Jungrak Choi, KAIST.

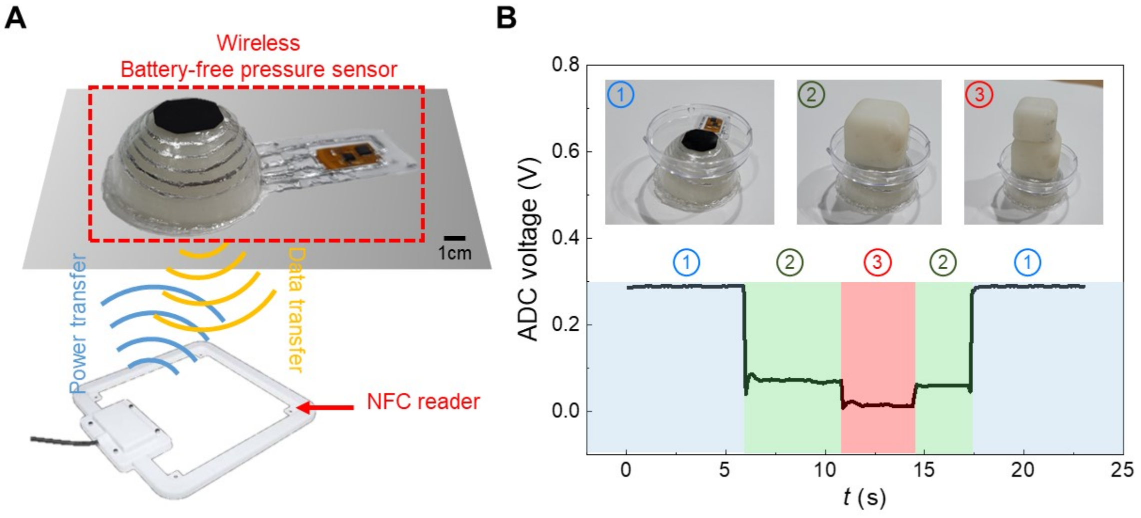

Fig. S10. Hemisphere shaped 3DE for wireless battery-free system with near-field communication (NFC). (**A**) Schematic illustration and photographs of the hemisphere-shaped pressure sensor. (**B**) Pressure sensor signal from the NFC reader while various weight of objects was put on the pressure sensor. Photo Credit: Jungrak Choi, KAIST.

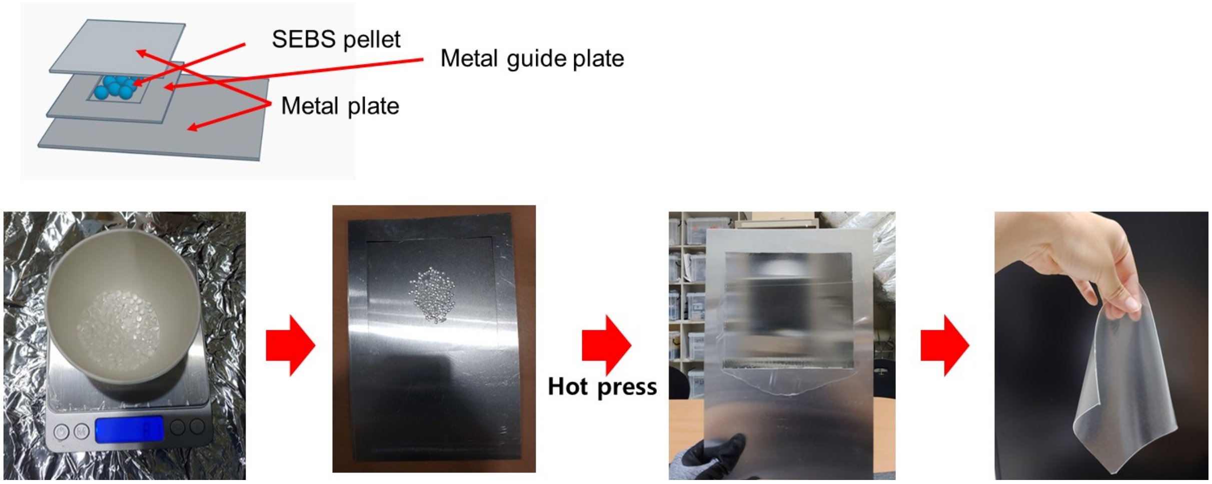

Fig. S11. SEBS film preparation. The SEBS pellet was measured and put into a square perforated plate (metal guide plate) and it was placed between bottom and top plates. Then, the plates were put into a pneumatic press machine and a pressure was applied to the plates for 5 minutes. After that, the plates were cooled down and the SEBS film was fabricated by detaching from the plates. Photo Credit: Jungrak Choi, KAIST.

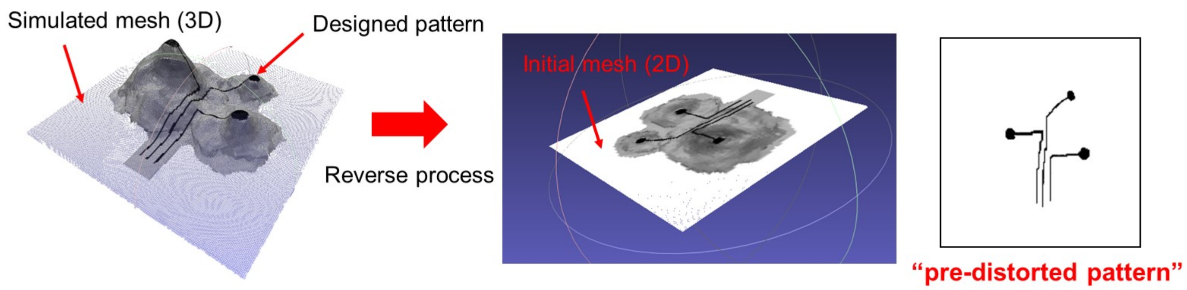

Fig. S12. Pre-distorted pattern generation process. Using the 3D mesh processing software, designed electronics pattern on the 3D model was projected onto the simulated mesh. From one-to-one correspondence between the initial mesh and simulated mesh, the pre-distorted pattern was obtained.

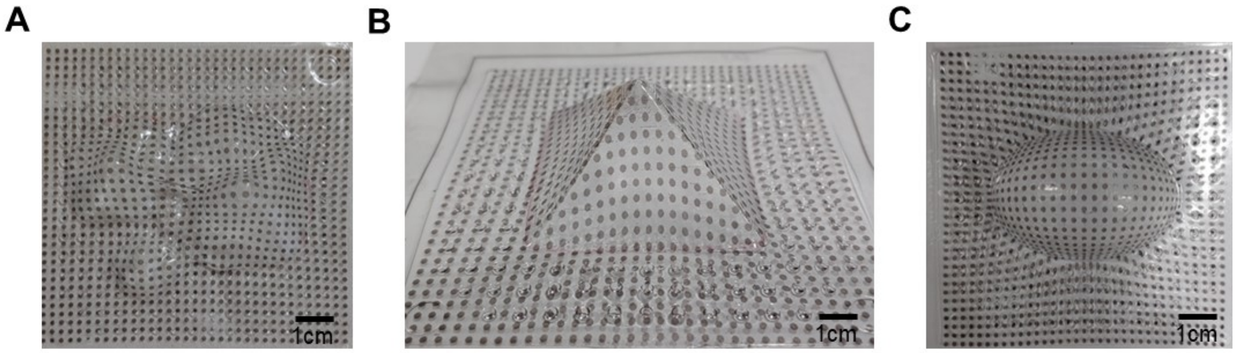

Fig. S13. Photographs of thermoformed SEBS film with distorted dot patterns. A periodic dot array pattern with a 3 mm pitch was printed on the 2D planar SEBS film. After thermoforming using different 3D molds such as a mountain (**A**), a pyramid (**B**), and a parabola (**C**), various thermoformed SEBS films with distorted dot patterns can be obtained. Photo Credit: Jungrak Choi, KAIST.

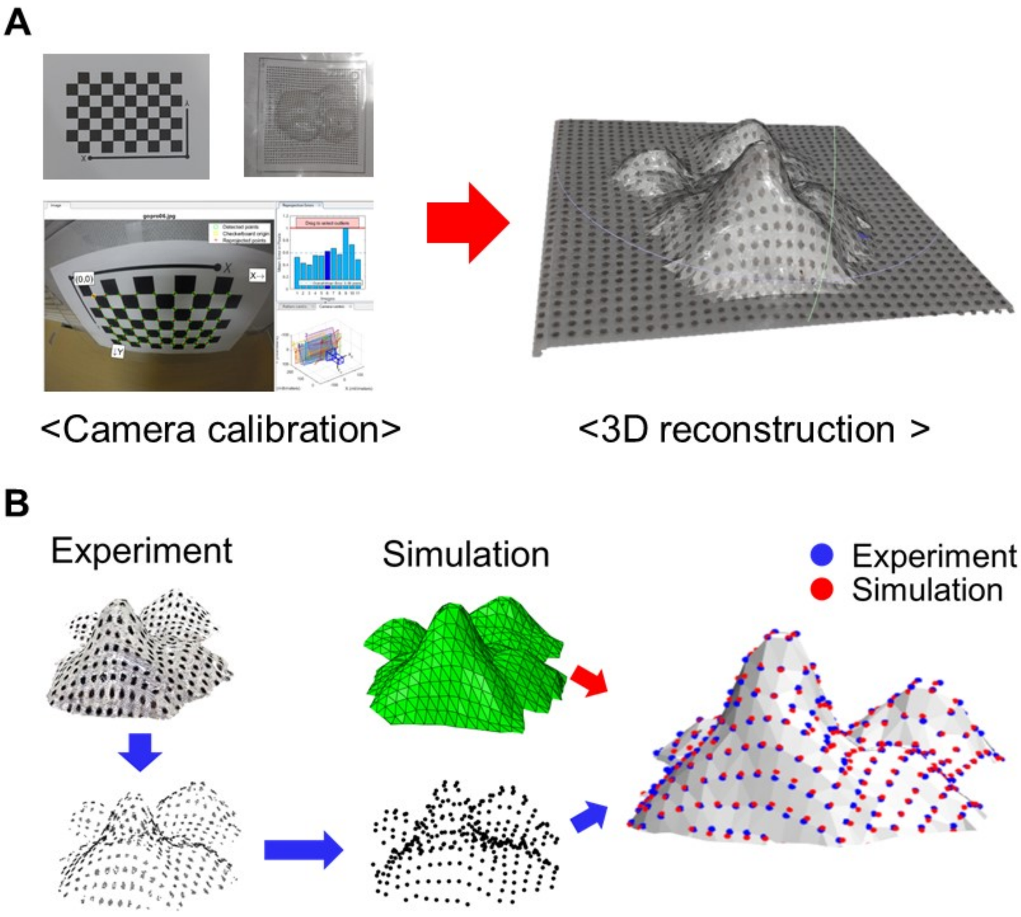

Fig. S14. 3D reconstruction of thermoformed SEBS film with distorted dot patterns. (**A**) 3D reconstruction of the thermoformed SEBS film using photogrammetry (i.e. 3D reconstruction from 2D photos taken at different angles with camera calibration). (**B**) Calculation process of distance between corresponding dots in the simulation and experimental results using the 3D mesh processing software and the numerical computing software. Photo Credit: Jungrak Choi, KAIST.

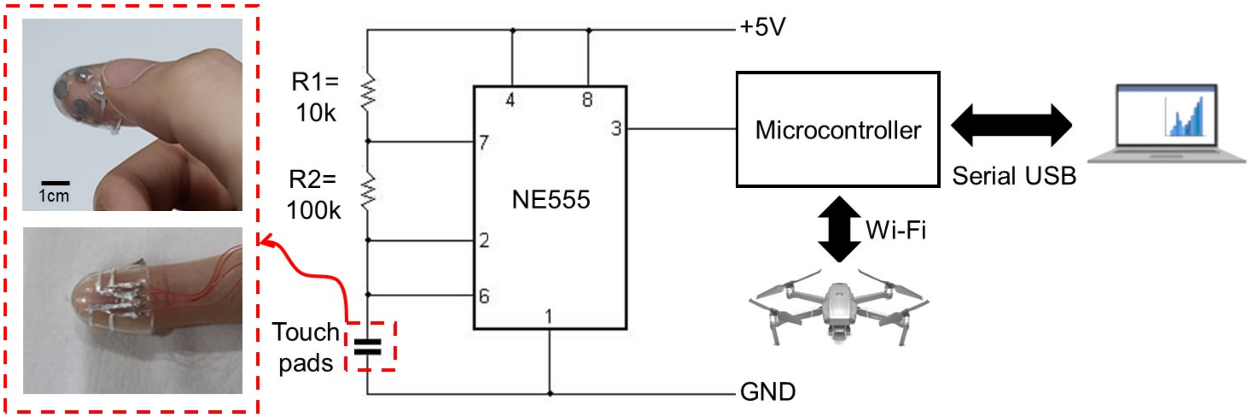

Fig. S15. Capacitance sensing circuit for fingertip-shaped capacitive touch sensor. The circuit was implemented with a NE555 timer IC and a microcontroller board. The microcontroller board measured the frequency and controlled a quadcopter drone via Wi-Fi communication. Photo Credit: Jungrak Choi, KAIST.

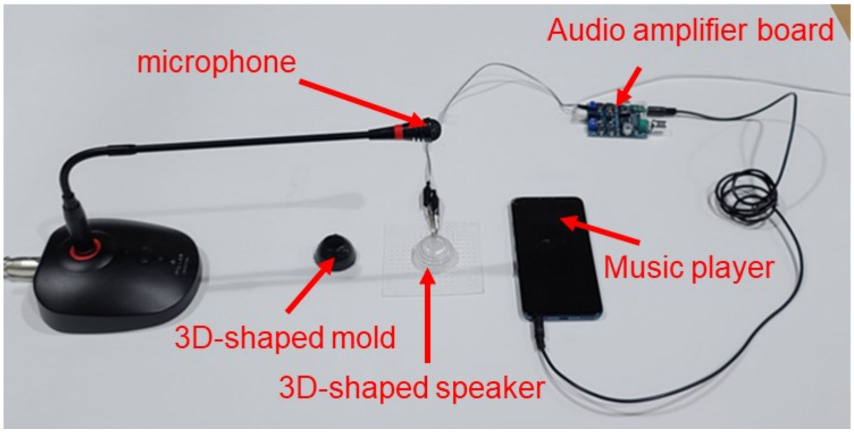

Fig. S16. Photograph of the system setup for demonstration of a hemisphere-shaped speaker. A sound from the 3D-shaped speaker was generated using an audio amplifier board. A sound from the 3D-shaped speaker was generated using an audio amplifier board connected to a commercial music player. While the music was played, the sound generated from the 3D-shaped speaker was recorded by using a commercial microphone. Photo Credit: Jungrak Choi, KAIST.

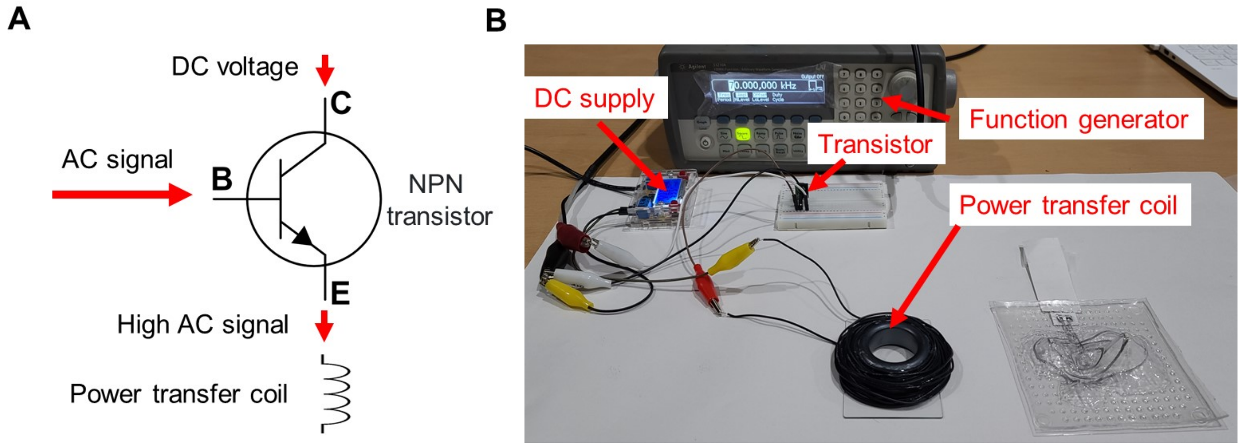

Fig. S17. Wireless power transmission system. (**A**) Schematic illustration of principle of the wireless power transmission system. (**B**) Photograph of the system setup. Function generator and NPN transistor were used to operate high AC signal. Function generator generated AC signals with a resonant frequency and the transistor amplified the signal to the power transfer coil. Photo Credit: Jungrak Choi, KAIST.

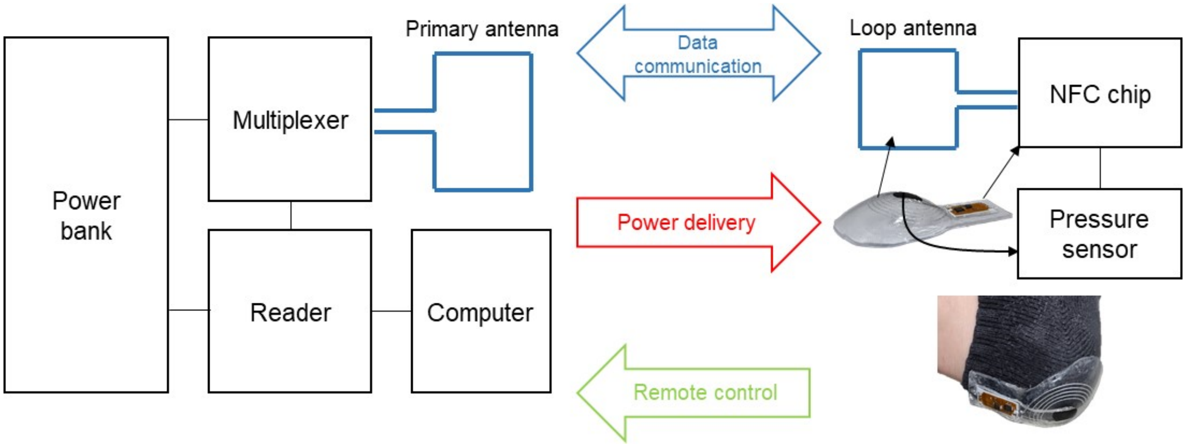

Fig. S18. Block diagram of NFC system. Pressure sensitive film and the 3D coil-shaped electrode (loop antenna) were connected to the NFC chip which was programmed to send the pressure data to the NFC reader via primary antenna. Photo Credit: Jungrak Choi, KAIST.

Table S1. Comparison of state-of-the-art fabrication process for 3D electronics (3DE).

Fabrication process	Ref.	Material	3D patterning method	Stretchability	Customizability	Printability on complex 3D structure*
3D printing	(*7*)	Ag nanoparticle based electrode + glass substrate	3 axis motor controller	x	O	O
	(8)	Ag/CNT ink based electrode + PET substrate	3 axis motor controller	x	O	O
	(*9*)	Ag/TPU based electrode + TPU substrate	N/A	O	O	x
Laser direct strucruing	(*10*)	Metal coated plastic film	Lens driver	x	O	O
	(*11*)	Metal coated fiber glass	Lens driver	x	O	O
Sparying	(*12*)	Ag nanoparticle based electrode + PI substrate	N/A	x	O	x
Hydrographic	(*13*)	Ag-In-Ga based electrode + PVA substrate	N/A	x	x	x
	(*14*)	Ag epoxy-liquid metal based electrode + PVA substrate	N/A	x	x	x
	(*15*)	Ag nanoparticle based electrode + PVA substrate	N/A	x	x	x
Thermoforming	(*17*)	Cu electrode + GFRP substrate	N/A	x	x	x
	(*18*)	Ag nanoparticle/flake based electrode + PET substrate	N/A	x	x	x
	(*19*)	Ag ink based electrode + PETG substrate	Iterative process with calibration sheet	x	O	O
	Our work	Liquid metal based electrode + SEBS substrate	Thermo-mechanical properties based simulation	O	O	O

*Printability on complex 3D structure means that the printed 3DE has both convex and concave shapes with various curvature radii.

Supplementary Videos:

● Movie S1 (.MP4 format). Electrical and mechanical stability test of the 3DE under stretching, twisting, and folding deformations.

● Movie S2 (.MP4 format). Thermoforming simulation process.

● Movie S3 (.MP4 format). Fingertip-shaped capacitive touch sensor signal.

● Movie S4 (.MP4 format). Fingertip-shaped capacitive touch sensor signal.

● Movie S5 (.MP4 format). Controlling quadcopter drone using fingertip-shaped capacitive touch sensor.

● Movie S6 (.MP4 format). Demonstration of mountain-shaped wireless power transmission system.

● Movie S7 (.MP4 format). Demonstration of 3D mogul-shaped wireless power transmission system.

● Movie S8 (.MP4 format). Demonstration of hemisphere-shaped wireless battery-free pressure sensor.

● Movie S9 (.MP4 format). Demonstration of heel-shaped wireless battery-free pressure sensor.
